# Penetrating Femoral Trauma: A Report on Its Management and Outcome

**DOI:** 10.7759/cureus.70776

**Published:** 2024-10-03

**Authors:** Konstantinos Zygogiannis, Dimitrios Kalatzis, Konstantinos Kaoullas, Dionisios Nikolopoulos, Georgios C Thivaios

**Affiliations:** 1 Department of Scoliosis and Spine, KAT General Hospital, Athens, GRC; 2 Department of Orthopedics and Traumatology, Laiko General Hospital of Athens, Athens, GRC

**Keywords:** femoral injury management, penetrating femoral trauma, soft tissue graft, trauma complications, traumatic vascular injury

## Abstract

Penetrating trauma of the femur poses significant life-threatening risks due to potential complications, particularly involving the femoral structures such as the femoral artery, femoral vein, and femoral nerve. These injuries can lead to severe outcomes, especially when accompanied by soft tissue damage. Effective management requires a thorough understanding of the injury’s nature, including the extent of soft tissue involvement and associated complications. This report presents a case of a 42-year-old male patient with a penetrating femoral injury through the anterior and posterior aspect of the lower limb without associated fracture, which highlights the importance of prompt surgical intervention and the use of flap reconstruction to address complications such as necrotic soft tissue.

## Introduction

Penetrating femoral trauma is a life-threatening situation for the patient as it may come with severe complications. The femoral vessels consist of the blood vessels that traverse the femoral triangle and enter the adductor canal, subsequently running along the thigh until they reach the area behind the knee. These major vessels include the femoral artery, commonly referred to as the common femoral artery in this region, and the femoral vein. Vascular trauma can statistically occur in approximately 3% of the total civilian and military injuries [1], in which extremity involvement may vary from 27% to 87% [2]. More specifically, civilian femoral vascular trauma may occur in 27-72% of all vascular trauma [3].

Another aspect to be considered in these injuries is the soft tissue damage. The extent of the approach for the surgical removal of foreign objects or the damage caused by the penetration force and the microvasculature's destruction can significantly affect limb salvage or surgical outcomes [4]. The clinical importance of distinguishing between closed and open soft-tissue injuries and understanding the incidence and impact of associated injuries, demographic factors, prehospital and in-hospital resuscitation efforts, surgical treatment approaches, and major complications has yet to be sufficiently defined [4]. Flap reconstruction for repairing such injuries can be categorized into local and free flap types. Generally, flaps are classified based on their blood supply, the location of the donor site, and the type of tissue being transferred [5].

## Case presentation

**Figure 1:**
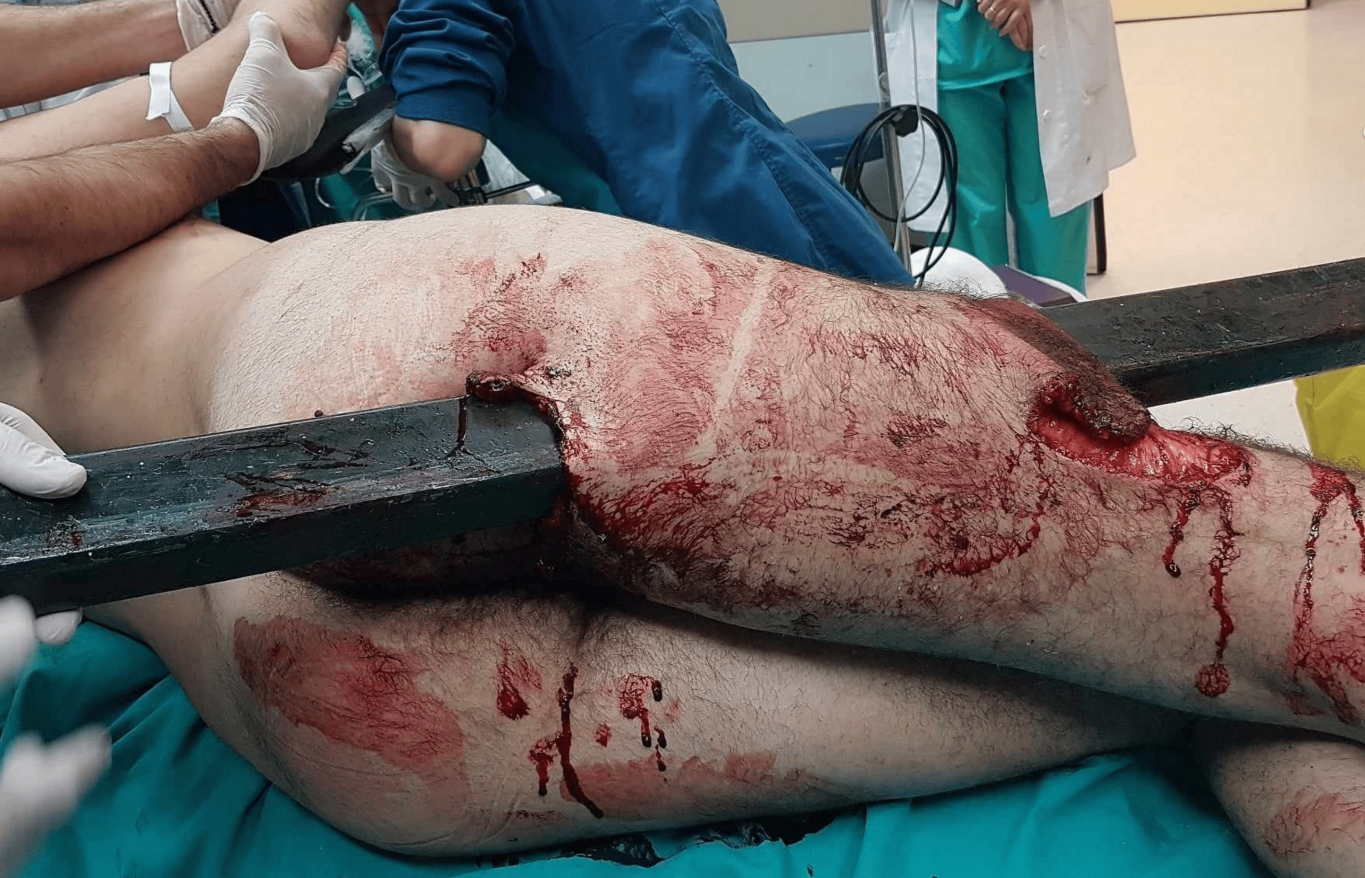
Patient with penetrating femoral trauma at the moment of arrival. The entry point of the foreign object was anterior to the femur while the exiting point was posterior.

A 42-year-old male sustained a penetrating femoral injury while falling from a height of 2 meters on to a metallic pillar. The patient arrived at the orthopedic emergency department with the metallic pillar still impaled, as shown in Figure 1. He was hemodynamically stable and neurologically intact. Notably, the X-rays did not reveal any evident fracture and the CT angiography did not show any major vessel injury.

**Figure 2:**
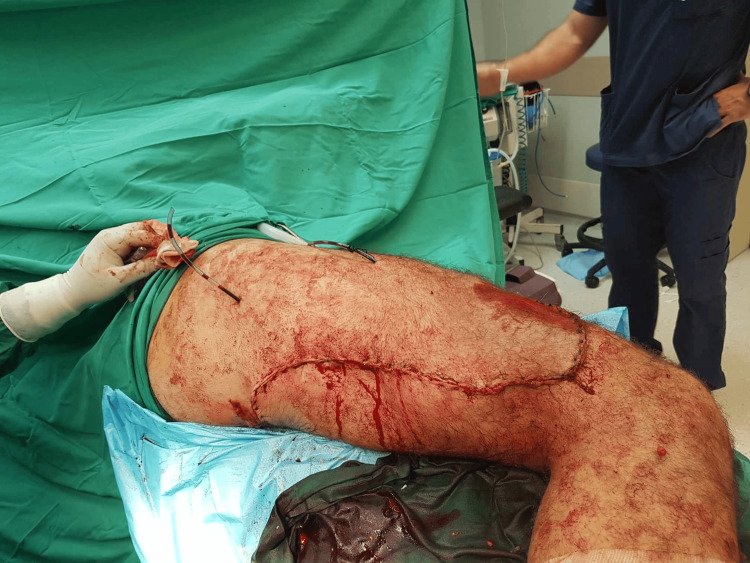
Immediate postoperative image of the femur after the open surgical removal of the foreign object. The incision was not a typical lateral because of the trajectory of the foreign object. An incision around the object was chosen for safe removal.

He immediately underwent an uneventful surgical removal of the foreign object through an extensive lateral femoral approach. An incision was made around the foreign object as seen in Figure 2. After the dissection of the subcutaneous fat, the fascia lata was incised with a scalpel and split with scissors parallel to the femur, along its fibers. Then the muscle fascia over the vastus lateralis was exposed. While lifting the vastus lateralis muscle fibers away from the intermuscular septum, extreme caution was taken when getting close to the femur. Perforating blood vessels should be ligated to avoid accidental tearing. In case of a vascular tear near the septum, there is a high chance of retraction into the back part of the thigh, which can sometimes lead to blood oozing. Next, blunt dissection was used to separate the vastus lateralis from the fascia lata. Afterward, it was retracted anteromedially. The main blood vessels and nerves are located on the inner side of the femur and were not exposed with this method.

**Figure 3:**
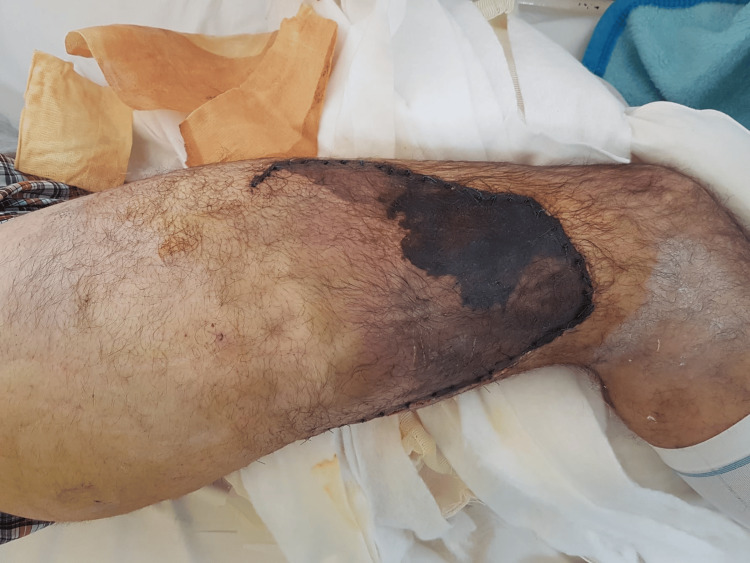
An area of skin necrosis observed on the 10th postoperative day at the distal part of the incision. There is no sign of puse leakage.

**Figure 4:**
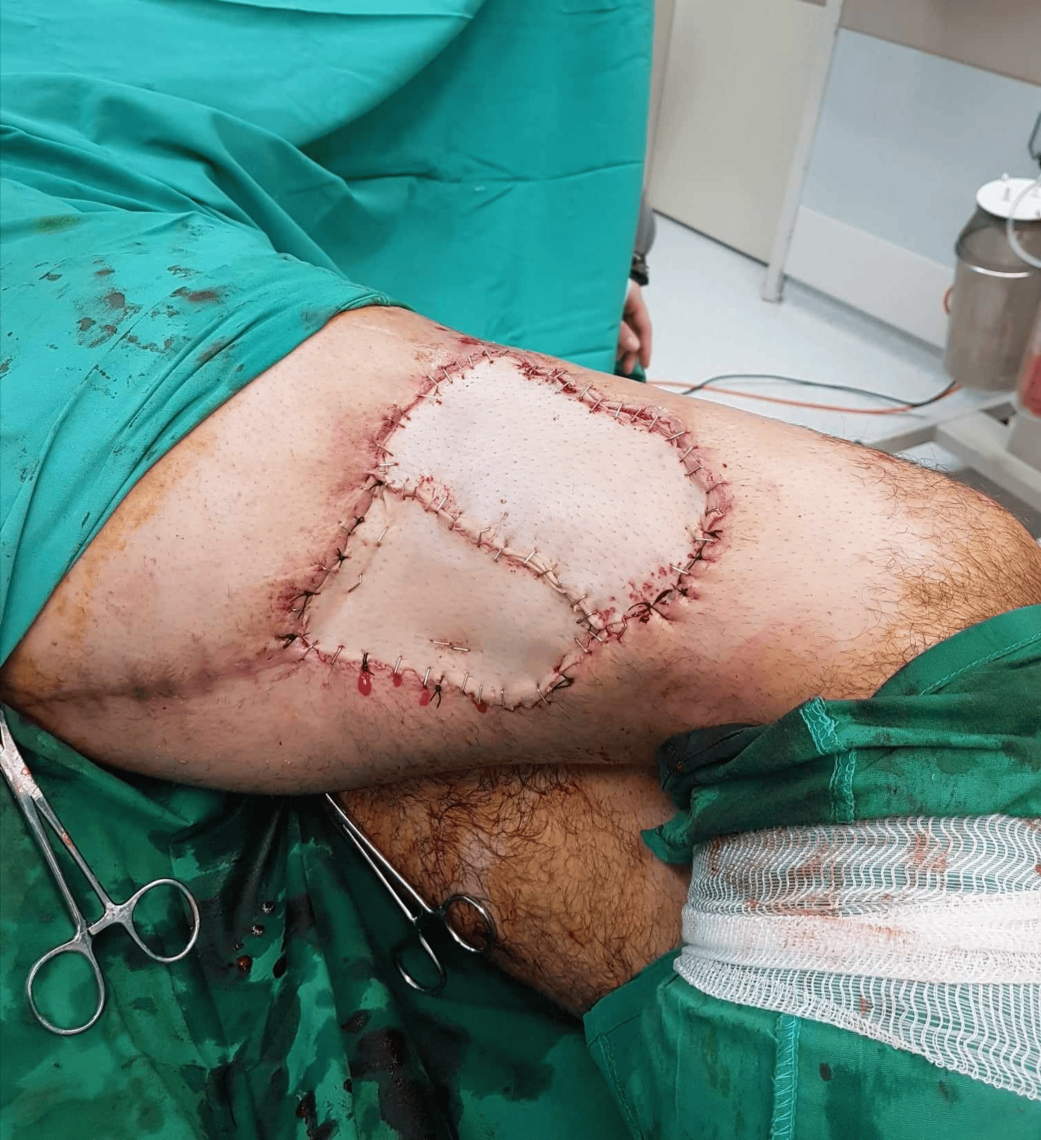
Postoperative image of free flap which was collected from the anteromedial area of the contralateral femur.

**Figure 5:**
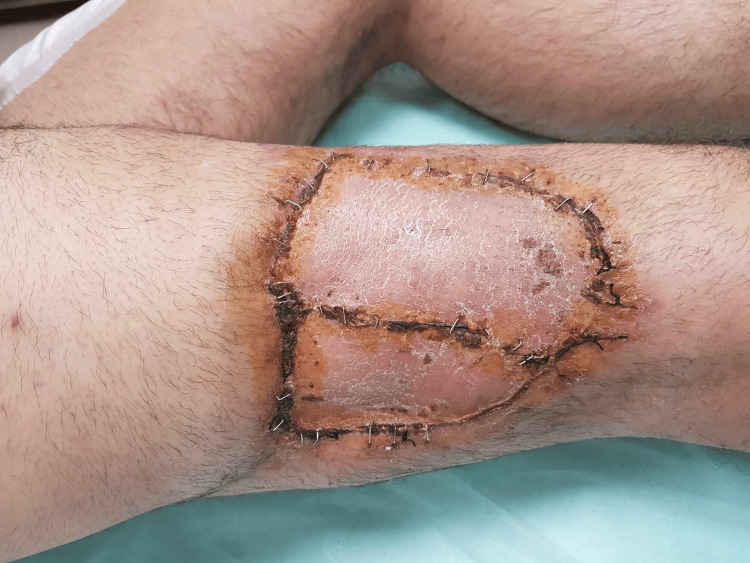
Free flap follow-up showing small areas of skin necrosis around the stitches but generally in good condition.

By the 10th postoperative day, while he was afebrile, he had developed an aseptic necrotic soft tissue area starting from the distal part of the incision and expanding proximally as shown in Figure 3. Since the intraoperative cultures were negative and the patient was afebrile, the plastic surgeons decided to treat this complication with a free flap as seen in Figure 4. The surgical trauma on the 45th postoperative day can be observed in Figure 5.

## Discussion

CT angiography (CTA)

Patients with abnormal physical examination often require immediate revascularization of the affected extremity. Diagnostic challenges may be present in cases where the clinical image does not reveal clear evidence to perform a CTA. Additionally, even when CTA reveals abnormal findings, surgical intervention is not always necessary [6]. Moreover, there is no widely accepted agreement on the specific indications for using CTAs in cases of limb trauma. This uncertainty has led to their overuse in some medical centers, potentially subjecting patients to avoidable risks [6]. In a retrospective cohort study, a total of 49 CTAs were analyzed, revealing vascular injury in 10 cases (20.4%) [7]. Statistically significant correlations with vascular injury were observed for the presence of hard signs (P < 0.001) and an Ankle-Brachial Pressure Index (API) below 0.9 (P = 0.02). Hard signs demonstrated a sensitivity of 90% and a specificity of 82%, while APIs exhibited both a specificity and sensitivity of 100%. Soft signs including a history of significant hemorrhage, decreased pulse compared to contralateral extremity, neurological impairment, and penetrating injury although displaying a sensitivity of 100%, were not significantly associated with vascular injury due to their low specificity [7]. 

Soft tissue

Free microvascular tissue transfer is currently recognized as a highly effective surgical technique for reconstructing complex skin and soft tissue defects. However, the failure or loss of a free flap through a possible infection, necrosis, or hernia remains a significant complication, often necessitating further surgeries, extended hospital stays, and increased healthcare costs [8]. Therefore, ensuring patient safety and minimizing risk are critical considerations in the indication and planning of such surgical procedures. Understanding the factors and causes that elevate the risk of flap failure is essential for implementing strategies to mitigate this risk. Numerous factors associated with compromised safety in free tissue transfer are discussed in the literature [9]. In the context of free flap surgery, perioperative factors such as anemia, preoperative hemoglobin levels, intraoperative blood loss, and the necessity of blood transfusion are critically important, alongside patient-specific characteristics. These factors significantly influence surgical outcomes and must be carefully managed to optimize the success of the procedure [10].

## Conclusions

Penetrating femoral trauma is a challenging surgical condition as the treatment may need a multidisciplinary approach. A high level of suspicion of underlying vascular pathology and knowledge of soft-tissue defect management can lead to successful management and outcomes.
